# Rapid Maxillary Expansion and Nocturnal Enuresis in Children and Adolescents: A Systematic Review of Controlled Clinical Trials

**DOI:** 10.1155/2021/1004629

**Published:** 2021-06-03

**Authors:** Khaled Khalaf, Dina Mansour, Zain Sawalha, Sima Habrawi

**Affiliations:** Department of Preventive and Restorative Dentistry, University of Sharjah, Sharjah, UAE

## Abstract

**Objectives:**

To evaluate the effectiveness of rapid palatal expansion in the treatment of nocturnal enuresis among 6–18-year-old children and adolescents.

**Methods:**

Comprehensive searches were carried out in 6 electronic databases (EBSCO, ProQuest, Clinical Key, Science Direct, SCOPUS, and OVID) and supplemented by additional manual searches in 4 orthodontic journals until June 2020. Randomized controlled clinical trials (RCTs) and controlled clinical trials (CCTs) of children and adolescents aged 6–18 years old of both genders who underwent rapid palatal expansion and were considered unresponsive to previous conventional nocturnal enuresis treatment were included in this review. Risk of bias of individual trials was assessed using the Risk of Bias in Non-randomized Studies of Interventions (ROBINS-I) assessment tool for CCTs and the revised Cochrane Risk-of-Bias tool for RCTs (RoB 2).

**Results:**

Four studies met all inclusion criteria and were finally included in this systematic review, of which one was an RCT and three were CCTs. Reduction in nocturnal enuresis frequency was reported in all included studies with varying rates and methods of reporting, but most studies reported a statistically significant reduction in the number of wet nights per week. The average range of becoming completely dry 1 year after treatment with an RME was 0%–60%. Also, there was a statistically significant correlation between an improvement in bedwetting and an increase in nasal volume after the use of RME.

**Conclusion:**

A rapid palatal expansion device may be considered as an alternative treatment option of the nocturnal enuresis condition with guarded prognosis when other treatment modalities have failed.

## 1. Introduction

Nocturnal enuresis (NE) or bedwetting (BW) is a prevalent condition that affects around 10% of children around the world, making it the second most common problem in school aged children after asthma and allergies [1–3]. It is defined as the involuntary voiding of urine by distinct acts of micturition at night in children 5 years old and above and is more common in males than females [4].

Monosymptomatic nocturnal enuresis is defined as the absence of or subtle daytime symptoms; presents 80% of cases with NE. Furthermore, cases can be classified into either primary (children who never achieved dryness) or secondary (dryness has been achieved for at least six months before enuresis begins) [5]. On the brighter side, NE is reported to have annual spontaneous cure rate of 15% [6]. Rarely, approximately in 0.5% to 2% of cases, NE persists in otherwise-healthy adults [7]. It is important to distinguish between NE and nocturia which is defined as the frequent night awakening to void [8].

The pathogenesis of NE is considered to be multifactorial and complex. However, previous studies were able to clear some ambiguities and highlight some important factors linked to this disorder. Historically, bedwetting was regarded a psychiatric disorder. Over the years, a clearer understanding has been obtained and causative factors have been narrowed down to mainly three reasons: (1) excessive production of urine at night, (2) hyperactivity of the bladder's smooth muscle, and (3) the inability to wake while asleep to empty the bladder when full. Furthermore, it has been reported in some patients that enuresis can be caused by a blockage of the upper airway [9].

Surprisingly, numerous “enuresis genes” have been detected making this disorder is highly hereditary, with astonishing increase in risk of 5–7% if one parent was affected [10]. Bedwetting is a stressful condition that has a negative impact on the child's quality of life during their development; such drawbacks can immensely improve with successful treatment [11].

Results obtained from multiple meta-analyses and clinical trials proposed different treatment modalities for children with NE [5, 9, 11, 12]. Such interventions usually start at the onset of the problem which is around 5–6 years of age. Generally, treatment options include motivational therapy, bladder training, fluid management, night alarms, and pharmacological agents such as desmopressin and tricyclic antidepressants. However, evidence is mostly in favor of the enuresis alarm which is considered the most effective and lasting management approach as has been shown in a systematic review of a large number of clinical trials (56). The second most accepted treatment option is the use of desmopressin or imipramine, an analogue of the vasopressin hormone, which reduces the production of urine and has been in use in the medical field for many years [11, 12].

The current treatment modalities provide results that are far from satisfactory and have been proven by various trials to have minimal efficacy in the management of this condition. Therefore, investigators have started focusing on alternative treatment options. According to several case reports, there seems to be a correlation between the resolution of upper airway blockage and an improvement in the condition of NE, since up to 80% of enuretic children have concurrent sleep apnea [13]. From this point, several other treatment options were described for the treatment of sleep disordered breathing and airway obstruction. The most common treatment proposed was adenotonsillectomy, to reduce nocturnal resistance airflow, therefore alleviating NE [14].

Some studies have reported the use of rapid maxillary expansion as a treatment modality to treat NE [14–16]. Expansion of approximately 5 mm was achieved by applying an orthodontic device to increase the maxillary width within 10–14 days [17, 18]. However, it is not yet known how effective this form of intervention to treat young children with NE who were unresponsive to the commonly used treatment modalities.

Therefore, the aim of our systematic review was to investigate whether a rapid maxillary expansion is an effective management approach in alleviating or treating nocturnal enuresis of young children and adolescents who were unresponsive to the commonly used treatment modalities.

## 2. Materials and Methods

### 2.1. Protocol and Registration

This systematic review was executed using the Prisma checklist guidelines and registered in PROSPERO (International Prospective Register of Systematic Reviews) under the registration number CRD42020170752.

### 2.2. Information Sources and Search Strategy

A comprehensive search strategy was performed using both manual and electronic sources to identify and include all potential articles. The electronic database search included the following databases: EBSCO (January 1990–June 2020), ProQuest (January 1998–June 2020), Clinical Key (October 1990–June 2020), Science Direct (June 1945–June 2020) SCOPUS (1990–2020), and OVID (1990–June 2020).

The manual search included the following journals: American Journal of Orthodontics and Dentofacial Orthopedics [AJODO] (July 1986–June 2020), Journal of orthodontics [JO] (March 2003–June 2020), Angle Orthodontist [AO] (January 1931–June 2020), and European Journal of Orthodontics [EJO] (February 1996–June 2020).

A combination of medical and non-medical terms were used for searching the electronic databases; terms included “rapid maxillary expansion”, “rapid palatal expansion,” “rapid expander”, “maxillary expansion”, “nocturnal enuresis”, “bedwetting”, and “children” and “adolescents”. Search terms were adapted to each electronic database to identify all potential articles indexed in the database.

### 2.3. Selection of Studies

Following a comprehensive search, only studies fulfilling the following criteria were included in this systematic review: (1) children and adolescents of either gender with 6–18 years old, (2) diagnosed with either primary or secondary therapy resistant NE, (3) had rapid maxillary expansion to treat nocturnal enuresis as the main outcome, and (4) were followed for at least six months post expansion. Papers that were reported in non-English or included participants with heavy snoring, sleep apnea, untreated constipation, concurrent urological, endocrinological, nephrological, odonatological or psychiatric disorders, and children and adolescents involved in ongoing enuretic treatment were excluded. Furthermore, study design was limited to only randomized controlled clinical trials and controlled clinical trials.

The PICOs components used in this systematic review were as follows:

  Population: children and adolescents of either gender aged 6–18 years old who were diagnosed with either primary or secondary therapy resistant NE.
  Intervention: rapid maxillary expansion device to expand the maxilla.
  Comparison: no expansion of the maxilla/passive rapid maxillary expansion device.
  Primary outcome measure: the number of wet nights per week.
  Secondary outcome measures: the number of responders, intermediate responders, and non-responders; cure rate (complete dryness) in the short-term (one month, 3 months and 6 months) and in the log-term (one year and 3 years); nasal volume after the use of RME.
  Study design: randomized controlled clinical trials (RCCTs) and controlled clinical trials (CCTs).

Titles, abstracts, and finally full texts of possible articles were scrutinized. Furthermore, references of the identified full text studies were inspected to identify additional ones for inclusion in the systematic review. The electronic and hand search was carried out in duplicate by two teams of investigators who met thereafter and had to agree. In the case of disagreement, a third author was consulted to reach a final decision.

### 2.4. Data Extraction

A form was created and used to extract all relevant information from the final included articles. The extraction of data was performed in duplicate to ensure accuracy of information gathered. For each article included, participant's demographic data, frequency of NE, signs and symptoms of upper airway obstruction (e.g., snoring, open mouth during sleep, and sleep apnea), Angle's classification, presence of crossbites, daily palatal expansion, duration of expansion, type of appliance used, expansion end point, study methodology, response rates (i.e., responders, partial responders, and non-responders), time to become completely dry or improved, follow-up periods and enuresis type (i.e., primary or secondary), and type of statistical analysis used (if any) were gathered (Table 1).

### 2.5. Risk of Bias of Individual Trials

Two investigators independently assessed the risk of bias of the included articles using suitable assessment tools. Any disagreement between the two investigators was settled by a third author. Non-randomized clinical trials were assessed using the Risk of Bias in Non-randomized Studies of Interventions (ROBINS-I) assessment tool [19]. On the other hand, randomized clinical trials were assessed using the revised Cochrane Risk-of-Bias tool for randomized trials (RoB 2) [20].

### 2.6. Data Synthesis Strategy

Data from the included studies will ideally be analyzed quantitatively using a meta-analysis if deemed appropriate, i.e., all included studies are homogenous in terms of study design and outcome measures reported and all are of low risk of bias; otherwise a descriptive (narrative) analysis will be carried out.

### 2.7. Assessment of Quality of Evidence Presented by This Review

Quality of evidence presented by this review was assessed using the GRADE approach (Grading of Recommendations Assessment, Development, and Evaluation) [21]. It consists of five assessment domains: risk of bias, inconsistency, imprecision, indirectness, and publication bias. A rating grade of high, moderate, low, or very-low is given to the quality of evidence presented by the review based on the above domains.

## 3. Results

### 3.1. Samples and Intervention Characteristics

**Figure 1:**
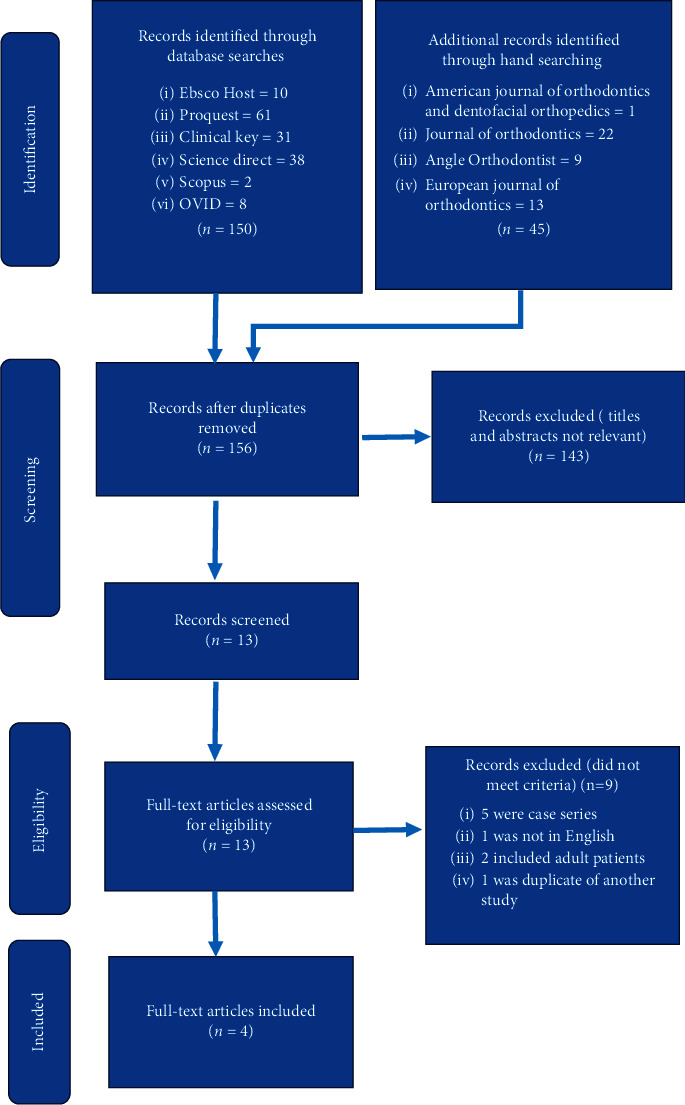
Flowchart of identifying, screening, and selecting suitable studies using Preferred Reporting Items for Systematic Reviews and Meta-Analyses (PRISMA).

Figure 1 shows the process of identifying and selecting all suitable articles for inclusion in this review. In total, 195 articles were assessed, of which 150 were from the online databases, and 45 from the manual search. Thirty-nine studies were duplicates, and 143 were not relevant from their abstracts and titles, thus leaving 13 articles deemed suitable at this stage for the inclusion in this review. Following probing the full texts of these studies, 9 were excluded, of which 5 were case series, 1 was not in English, 2 included adult patients, and 1 was duplicate of another study. Thus, only four studies were finally included in this systematic review, of which one was a randomized clinical trial (RCT) and three were non-randomized clinical trials (CCTs). One of these CCTs was published as two separate articles and thus were considered as one study in this systematic review. Two of the three CCTs used the expansion appliance as a placebo and one study had a separate control group.

The agreement between the reviewers regarding searching, identifying, and selecting the final studies was assessed using a kappa statistic which was found to be 0.89.

In total, the number of participants in the studies included was 129 children and adolescents (3 dropped out) with an age range of 6 to 18 years. Descriptive and demographic data are presented in Table 1. All studies used a hyrax screw which was inserted in an acrylic expanding device; some studies used a sham device for placebo effect. Expansion was achieved by applying a rapid heavy force delivered to the mid palatal suture by turning the screw twice/day. Expansion caused the suture to distract and the two palatal shelves to be pushed apart causing a diastema between the central incisors [22]. The expansion device was kept in place for a few months after finishing the active phase (10–14 days) as a retainer and to prevent further collapse.

### 3.2. Risk of Bias within Studies

**Figure 2:**
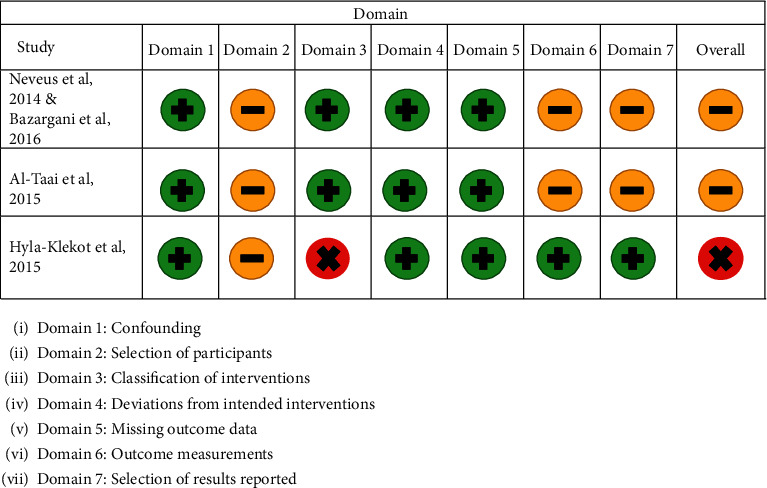
Risk of bias assessment for the non-RCT studies included in the review.

**Figure 3:**
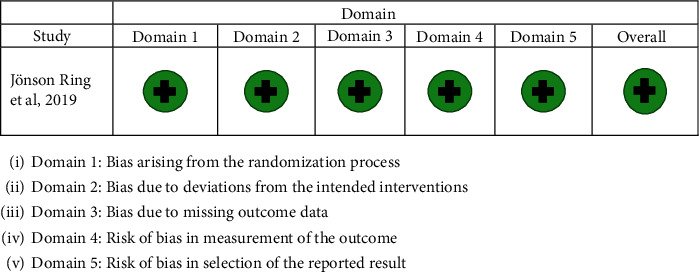
Risk of bias assessment for the RCT study included in the review.

Figures 2 and 3 show the risk of bias judgement for the final studies with the tools used and a justification for the grade given for each study. Two of the three CCTs [23–25] were assessed at moderate risk of bias and one at serious risk of bias [27], whereas the RCT [26] was assessed to be at low risk of bias.

### 3.3. Results of Individual Studies

Results of the individual studies will be summarized and reported narratively as it was not possible to pool the findings in a meta-analysis approach due to the heterogeneity among the included studies in terms of study design and outcome measures reported and all but one study being of at least moderate risk of bias.

#### 3.3.1. Improvement in Nocturnal Enuresis

Reduction in nocturnal enuresis was reported in all included studies with varying rates and methods of reporting such an improvement. Three studies [23, 24, 27] reported a reduction in NE frequency and presented their findings in terms of responders (patients who became completely dry), intermediate responders (patients who still wet the bed occasionally), and non-responders (patients who did not improve and wet the bed regularly). Ring et al. [26] reported a statistically significant decrease in the number of wet nights during 2 weeks following the treatment with an RME (*p* < 0.001), but no significant decrease was found following the placebo treatment (*p* > 0.40). The mean number of wet nights per 2 weeks has significantly declined from 11.9 to 8.5, which was translated as one full responder, 10 intermediate responders, and 20 non-responders. However, the difference between the intervention and control groups was not statistically significant [26]. On the other hand, Bazargani et al. [23] and Nevéus et al. [24] reported a statistically significant decrease in NE following the treatment with an RME (*p* < 0.001) with a reduction of number of wet nights per week from 5.48 ± 1.48 at baseline to 3.09 ± 2.49 after RME. This represented as 48.5% of the patients to be full or intermediate responders and 51.5% of the patients considered as non-responders.

Al-Taai et al. [25] did not use the previously mentioned terms of full, intermediate, and non-responders; instead, he reported that after RME expansion six out of 12 patients showed a complete dryness, and the remaining 6 patients showed an improvement in the NE. On the contrary, the control group (7 patients) showed no significant change in the frequency of their NE (*p* > 0.05).

Finally, Hyla-Klekot et al. [27] described the intensity level of NE using a 4-grade scale, where a score of 4 = bedwetting twice a night, 3 = once a night, 2 = once or twice a week, and 1 = once or twice a month. After the RME treatment, 10/16 patients were completely dry, and this remained so 3 years later. 5/16 patients had their frequency decreased by one or two grades and 1 child did not improve at all.

#### 3.3.2. Nasopharyngeal Airway Changes

Bazargani et al. [23] and Nevéus et al. [24] obtained a polysomnographic registration along with rhinomanometry and acoustic rhinometry to measure nasal airway patency, airflow, and oxygen saturation. They demonstrated a significant increase in nasal volume and airflow after treatment with the RME (*p* = 0.012). In addition, they reported a statistically significant association between a decrease in the enuresis and an increase in nasal volume (*p* = 0.034), but they could not detect such an association between a reduction in the enuresis and an increased nasal airflow (*p* = 0.46) [23]. Furthermore, Nevéus et al. [24] reported a resolution of the snoring habits as well as a greater nasal volume in the individuals who were treated with the RME.

Al-Taai et al. [25] further investigated airway dimensional changes using a coronal section of computed tomography (CT) scan of the sinuses as well as anterior rhinometry measurements to assess nasal airflow and resistance. They concluded that nasal airflow increased significantly (*p* < 0.001) with nasal airflow rising from 405.05 cm3/s before the expansion to 584.86 cm3/s following the expansion. The CT scans taken also showed a significant increase (*p* < 0.001) in the width of the nasal cavity at the level of the inferior concha and a significant decrease (*p* < 0.001) in the nasal airway resistance after the expansion with the RME compared to prior to the expansion.

#### 3.3.3. Psychological Impact and Sleep Disorders

Most of the studies highlighted that persisting NE was a high risk of psychosocial comorbidity and negatively affects the quality of life. The feeling of helplessness of enuretic patients highlights the magnitude and complexity of the problem. Persisting enuresis adversely affects the coping, social competence, and school performance of enuretic patients when compared to their normal peers. Furthermore, a negative correlation exists between the self-esteem of an enuretic child and the chance of treatment failure [12, 23, 26].

Furthermore, a considerable number of enuretic patients was found to have concurrent sleep problems, including but not limited to snoring and sleep apnea. Elimination of airway obstruction at nasopharyngeal or oropharyngeal level with either tonsillectomy or adenoidectomy or both may improve the nocturnal enuresis, and it showed favorable results [9, 23, 25].

It is worth mentioning that many of our included studies have excluded patients who have any concurrent urological, endocrinological, nephrological, odonatological, or psychiatric disorders.

#### 3.3.4. Expansion and Retention

The expansion device used in our included studies was the RME expansion device with hyrax screw soldered to orthodontic bands on the upper permanent first molars. However, Al-Taai et al. [25] also applied bands on the first premolars or second primary molars of patients who had an unerupted first premolars.

Retention after expansion was done by leaving the same appliance in situ for a few months [23, 24] or by using a Hawley retainer [25]. However, Hyla-Klekot et al. [27] did not elaborate on the method of expansion and retention.

Follow-up period ranged from 6 months to 3 years. At least 6 months of follow-up was required post expansion for a study to be included in our systematic review. The follow-up assessment was done by either phone or direct interviews [24].

#### 3.3.5. Occlusion

Investigating changes in occlusion brought about by RME devices was not the primary objective in the studies included in this systematic review. However, most studies obtained dental casts to check occlusion along with intermolar, interpremolar, and intercanine distances. They revealed that occlusion characteristics did not affect the outcome. Furthermore, it was found that the RME device can be used as an alternative method to improve the NE in patients with a normal bucco-lingual relationship of the posterior teeth with no detriment to the occlusion [23].

It was noticeable that reporting changes in occlusion was not consistent between studies. Hyla-Klekot et al. [27] reported the percentages of malocclusion and unilateral or bilateral crossbites in the patients included in the study. They showed that the most common malocclusion was a class II (35%) which was often associated with the presence of a deep bite (33%), whereas a posterior crossbite was reported in 14% of the individuals. The least common malocclusions were the class III (4%) and the open bite (2%). They concluded that the main aim of RME treatment was not to correct the malocclusion but to only reduce the NE. Similarly, Bazargani et al. [23] reported percentages of malocclusions and crossbites. Only two of the 34 subjects included in their study had posterior crossbites; 26 patients (76%) had an Angle Class I, which included the two crossbite cases; 7 (21%) had an Angle Class II with a mean overjet of 5.6 mm; and 1 (3%) had an Angle Class III. They concluded that no untoward impact could be observed on the occlusion in the long-term, thus corroborating the above finding that patients with normal occlusal features can be treated with a rapid maxillary expansion to improve their NE condition. Al-Taai et al. [25] reported patients with different degrees of crowding and only 2 out of 19 patients had a crossbite. They did not report on the skeletal class or Angle classification of their sample.

Such a variation in reporting occlusal changes was expected since the primary focus of the studies included in this review was the effect of an RME on the nocturnal enuresis.

### 3.4. Evaluating the Strength of Evidence Provided by This Review

The overall quality of evidence provided by this review for the main outcome measure, i.e., a decrease in the number of wet nights/week following the treatment with an RME device, was found to be very low. This was due to the moderate to critical risk of bias across the included studies except one, which was of low risk of bias, small sample sizes investigated by the majority of studies, and non-significant findings from a clinical point of view (Table 2).

## 4. Discussion

NE is a stressful condition that affects the child's emotional wellbeing immensely which further reflects on their quality of life, self-esteem, and school performance; such drawbacks can significantly improve with a successful treatment [11]. It has been reported that NE have an annual spontaneous cure rate of 15% [6] and up to date, there is no clinically approved treatment modality for the NE condition in patients, where all commonly used treatments can only produce slight improvement and thus are considered of minimal efficacy. Therefore, investigators have started focusing on alternative treatment options. One of the possible suggested causes of NE is an upper airway obstruction [9]. Moreover, up to 80% of enuretic patients have concurrent sleep apnea [13]. Thus, it was only logical to consider palatal expansion as a potential solution to NE in young patients.

In this systematic review, we found that the most commonly used and effective device for expansion is the RME device with a hyrax screw soldered to orthodontic bands on the first permanent molars. This orthodontic device results in expansion of the maxilla due to separation of the mid palatal suture over a period of 10–14 days where the midline screw was activated twice daily to achieve a total daily expansion of 0.5 mm [23–27]. The endpoint of expansion was concluded as when the occlusal surfaces of the palatal cusps of the upper first permanent molars came into contact with the occlusal surfaces of the buccal cusps of the lower first permanent molars [23–26].

Although examining the effect of RME on occlusion was not a primary objective in our review, it is worth mentioning that the RME device can be used as an alternative method to improve the NE condition in patients with a normal bucco-lingual relationship of the posterior teeth with no detriment to the occlusion [23]. Furthermore, the findings of the included studies show that the type of malocclusion does not bare any effect on the improvement of NE using RME devices. This means that the RME treatment modality, if it were proved to be effective in curing NE in patients, can be adopted as an alternative treatment option in young patients who suffer from NE and did not respond to the conventional management options regardless of the features of their occlusion.

A reduction in the nocturnal enuresis following the use of an RME device was reported in all included studies with varying rates and methods of reporting such an improvement. The average rate of becoming completely dry 1 year after the treatment with an RME device was found in the range of 0–60%. The results of this systematic review somehow agree with the findings reported by a previous systematic review [22] which concluded that a rapid palatal expansion in the management of NE in patients had a success rate of 31% (average rate of becoming completely dry 1 year after the treatment with an RME) and thus might be contemplated when other management approaches did not succeed. However, the latter study [22] provided a weak scientific evidence due to the inclusion of only inherently low-quality study types, i.e., case series, very limited search of electronic databases (Pubmed and Embase and additional articles from Google scholar) with no additional search of relevant journals and the grey literature, whereas the current systematic review represents the current literature more accurately since (1) it included only randomized and nonrandomized clinical trials providing a higher level of scientific evidence and (2) identified all relevant articles using multiple online databases and further expanded the search including hand searching of four different orthodontic journals and the grey literature.

The results of this systematic review may support the use of RME devices in the treatment of NE condition as a viable option when commonly used treatment modalities have failed for the following reasons: (1) a short active treatment duration of 10–14 days, (2) RME devices are considered minimally invasive compared to other treatment modalities, (3) RME devices can be used to correct pre-existing transverse occlusal discrepancies such as unilateral or bilateral crossbites, and (4) RME devices are well tolerated by patients with minimal side effects.

### 4.1. Strengths and Limitations

To our knowledge, the current systematic review is the most up-to-date review on the topic, conducted according to the PRISMA guidelines and presents the best available evidence in the literature. We also followed meticulous and strict inclusion/exclusion criteria to ensure that we solely studied the effect of the RME treatment approach on the NE condition minimizing confounding variables like systemic diseases. We limited our inclusion criteria of study designs to only RCTs and CCTs to minimize biases inherently committed in other study types. Furthermore, two of the studies included in our systematic review [23, 24] reported the use of polysomnographic registration along with rhinomanometry and acoustic rhinometry to measure nasal airway patency, airflow, and oxygen saturation in contrast to the previous systematic review by Poorsattar-Bejeh Mir et al. [22] which lacked studies that performed polysomnographic registration.

One of the limitations in our systematic review was the lack of a parallel control group in 2 of the included studies. Another limitation was that the overall sample size of participants was low. Moreover, some information, such as familial history of enuresis, exact and average bedwetting per night and per week, was not reported in all studies. Furthermore, limiting the language to the English was a limitation, but it is unlikely that a high quality article would have been published in a non-English language. Finally, it was not possible to synthesize the data of all included studies quantitatively using a meta-analysis due to the heterogeneity among the included studies in terms of study design and outcome measures reported and all but one study being of at least moderate risk of bias.

## 5. Conclusion

The use of RME in patients with NE resulted in a significant reduction of wet nights per week compared with no intervention, but the difference between the two groups was not statistically significant. More well-designed RCTs are required to form a definitive conclusion.

## Figures and Tables

**Table 1:** Summary data of the included studies.

Author, year of study	Study type	Participants	Inclusion and exclusion criteria	Intervention/control	Amount of expansion	Follow-up periods	Findings of the study
Neveus et al., 2014 and Bazargani et al., 2016 (one study published as two separate articles)	Nonrandomized controlled trail	(i) 34 (29 M, 5 F), one dropped out	(i) Inclusion criteria: children did not respond to conventional treatments	RME appliance activated for all patients/RME appliance was left passive for the initial 4 weeks for all patients	0.5 mm daily (0.25 mm morning, 0.25 mm night)	(i) Baseline (before treatment)	(i) The number of wet nights/week on the 4 follow-up periods was 5.48 ± 1.48, 5.12 ± 1.73, 3.09 ± 2.49, and 2.63 ± 2.81; *p* < 0.001
		(ii) Age: 8–15 years	(ii) Exclusion criteria: known general medical conditions or medications that are linked to NE			(ii) With the orthodontic appliance in situ	(ii) After RME the number of responders and intermediate responders was 16/33 (48.5%), and the number of nonresponders was 17/33 (51.5%)
						(iii) 6 months (after completion of expansion)	(iii) The long-term cure rate after 1 year was 18/30 (60%), whereas 12/30 (40%) had no long-term response
						(iv) 1 year post treatment	(iv) Nonresponders had more frequent enuresis (6.29 ± 1.31 versus 4.63 ± 1.15 wet nights/week; *p* = 0.001)

Al-Taai et al., 2015	Nonrandomized controlled trial	(i) 19 (1 M, 18 F)	(i) Inclusion criteria: healthy children with monosymptomatic primary NE (MPNE) treated with Minirin without long-term improvement	RME appliance activated for all patients/RME appliance was left passive for the initial 30 days for 7 patients	0.45 mm per day	(i) 2–3 months after RME	(i) The mean value of wetting per night before expansion = 2.21
		(ii) Age: 6–15 years	(ii) Exclusion criteria: dryness >6 months, known general medical conditions or medications that are linked to NE			(ii) 1 year after RME	(ii) The mean value of wetting per night 2–3 months after RME = 0.42
						(iii) 3 years after RME	(iii) 30 days after RME expansion, 6 out of 12 children demonstrated complete dryness, and the remaining demonstrated an improvement of NE
							(iv) No significant impact on NE (*p* > .05) was found in the control group (7 patients) 30 days after the use of a passive RME device
							(v) After 3 years, all patients reported complete dryness
Hyla-Klekot et al., 2015	Nonrandomized control trial	(i) 41 in total	(i) Inclusion criteria: present NE, lack of disease in the kidneys and urinary tract system	RME activated (16)/No RME (25)	Total of 6.5 mm	(i) Every month during the first 12 months	(i) 10/16 children in the intervention group did not wet the bed at all after 3 months and this was maintained 3 years later (8/16 children remained dry)
		(ii) Age: 6–18 years	(ii) Exclusion criteria: active dental caries, bad oral hygiene, inadequate number of teeth for fitting the appliance, and lack of cooperation with orthodontic treatment			(ii) 3 years	(ii) After 3 years, 50% of the children in the intervention group were completely dry compared with only 32% in the control group
		(iii) 16 experimental (9 M, 7 F)					(iii) After 3 years, there was 4.5 times increase in the reduction of NE in the experimental group compared with the control group
		(iv) 25 control (15 M, 10 F)					

Jönson Ring et al., 2019	Randomized clinical trail	(i) In total 38, 2 dropped out from the placebo group, age: 10.2 ± 1.8	(i) Inclusion criteria: primary NE with at least 7 wet nights fortnightly and nonresponders to first-line treatment	RME appliance activated for 2 weeks/RME appliance was left passive for the 2 weeks	0.5 mm per day	(i) Baseline (T0)	(i) From T0 to T1, the experimental group demonstrated a significant reduction of wet nights (mean difference = −2.2) and the placebo group demonstrated no significant reduction of wet nights, mean difference = −0.6). The difference between the 2 groups was not statistically significant
		(ii) Intervention group 18 (18 M), age (10.3 ± 1.8)	(ii) Exclusion criteria: known general medical conditions or medications that are linked to NE			(ii) 2 weeks (T1)	(ii) The mean reduction of wet nights for the whole group 6 months after expansion was significant (mean difference = −3.2)
		(iii) Placebo group 20 (17 M, 3 F), age (10.2 ± 1.8)				(iii) 6 months (T3)	(iii) 11 patients (35%) had a reduction in the frequency of NE by >50%
							(iv) At 6 months, the number of full, intermediate, and nonresponders was 1, 10, and 20, respectively
							(v) A wide maxilla and great voided volumes at baseline may be associated with a reduced frequency of enuresis

M: male, F: female, NE: nocturnal enuresis, RME: rapid maxillary expansion, NE: nocturnal enuresis.

**Table 2:** Rating the overall quality of evidence according to the GRADE's approach.

No. of participants	Risk of bias	Indirectness	Imprecision	Inconsistency	Publication bias	Overall quality of evidence
A reduction in the number of wet nights per week						
129	Serious^a^	Not serious^b^	Serious^C^	Not serious^d^	Not suspected^e^	Very low⊕〇〇〇

^a^Two non-RCTs were ranked of moderate ROB and one non-RCT was ranked of serious ROB. ^b^All included studies were similar in terms of the inclusion criteria of participants, interventions (RME), and the primary outcome measures (the number of wet nights per week). ^c^The total number of participants for the primary outcome was very small (129). In addition, although the best quality study [26] reported a statistically significant decrease in the number of wet nights/week during the 2 weeks following the treatment with an RME, the difference between the intervention and control groups was not statistically significant. ^d^All studies reported a similar pattern and magnitude of effect in the main outcome measure between the intervention and control group. ^e^A very comprehensive search of multiple sources was carried out. No clinical trials had been found to be registered in trials registry websites, but have not been published. Studies of positive and negative findings were published and included.

## Data Availability

This is a systematic review study. All relevant data are presented in the manuscript. However, any required further information can be provided by the corresponding author.
